# Bayesian Molecular Dating Analyses Combined with Mutational Profiling Suggest an Independent Origin and Evolution of SARS-CoV-2 Omicron BA.1 and BA.2 Sub-Lineages

**DOI:** 10.3390/v14122764

**Published:** 2022-12-12

**Authors:** Naveen Kumar, Rahul Kaushik, Ashutosh Singh, Vladimir N. Uversky, Kam Y. J. Zhang, Upasana Sahu, Sandeep Bhatia, Aniket Sanyal

**Affiliations:** 1 Diagnostics & Vaccines Group, ICAR-National Institute of High Security Animal Diseases, Bhopal 462022, India; 2 Biotechnology Research Center, Technology Innovation Institute, Abu Dhabi P.O. Box 3692, United Arab Emirates; 3 Center for Biosystems Dynamics Research, Laboratory for Structural Bioinformatics, Yokohama 230-0045, Japan; 4 Department of Molecular Medicine, Morsani College of Medicine, University of South Florida, Tampa, FL 33612, USA; 5 Federal Research Center ‘Pushchino, Scientific Center for Biological Research of the Russian Academy of Sciences’, Institute for Biological Instrumentation of the Russian Academy of Sciences, 142290 Pushchino, Russia

**Keywords:** COVID-19, SARS-CoV-2 Omicron, tMRCA, evolutionary rate, mutational profiling, selection pressure

## Abstract

The ongoing evolution of severe acute respiratory syndrome-coronavirus-2 (SARS-CoV-2) has resulted in the recent emergence of a highly divergent variant of concern (VOC) defined as Omicron or B.1.1.529. This VOC is of particular concern because it has the potential to evade most therapeutic antibodies and has undergone a sustained genetic evolution, resulting in the emergence of five distinct sub-lineages. However, the evolutionary dynamics of the initially identified Omicron BA.1 and BA.2 sub-lineages remain poorly understood. Herein, we combined Bayesian phylogenetic analysis, mutational profiling, and selection pressure analysis to track the virus’s genetic changes that drive the early evolutionary dynamics of the Omicron. Based on the Omicron dataset chosen for the improved temporal signals and sampled globally between November 2021 and January 2022, the most recent common ancestor (tMRCA) and substitution rates for BA.1 were estimated to be that of 18 September 2021 (95% highest posterior density (HPD), 4 August–22 October 2021) and 1.435 × 10^−3^ (95% HPD  =  1.021 × 10^−3^ − 1.869 × 10^−3^) substitution/site/year, respectively, whereas 3 November 2021 (95% highest posterior density (HPD) 26 September–28 November 2021) and 1.074 × 10^−3^ (95% HPD  =  6.444 × 10^−4^ − 1.586 × 10^−3^) substitution/site/year were estimated for the BA.2 sub-lineage. The findings of this study suggest that the Omicron BA.1 and BA.2 sub-lineages originated independently and evolved over time. Furthermore, we identified multiple sites in the spike protein undergoing continued diversifying selection that may alter the neutralization profile of BA.1. This study sheds light on the ongoing global genomic surveillance and Bayesian molecular dating analyses to better understand the evolutionary dynamics of the virus and, as a result, mitigate the impact of emerging variants on public health.

## 1. Introduction

Since the inception of the coronavirus disease 2019 (COVID-19) pandemic caused by severe acute respiratory syndrome-coronavirus-2 (SARS-CoV-2) in December 2019, multiple variants have emerged and rapidly spread, resulting in significant changes in SARS-CoV-2′s global evolutionary dynamics. The current nomenclature schemes for these variants are the Global Initiative on Sharing All Influenza Data (GISAID) [1], Pango [2], and Nextstrain [3]. Nonetheless, the World Health Organization (WHO) has classified these variants into Variants of Interest (VOIs) and Variants of Concern (VOCs), which makes it easier for their tracking, research, and, eventually, guiding the ongoing COVID-19 pandemic response on a global scale [4]. The former carries some peculiar genetic changes that may affect the COVID-19 severity, transmissibility, and ability to escape from existing diagnostic and therapeutic approaches, resulting in an emerging risk to global public health with increased community transmission. The latter has been shown to carry one or more genetic changes that directly affect global public health, such as enhanced virulence and/or transmissibility, resulting in a significant change in COVID-19 epidemiology and clinical disease severity, and reduced effectiveness of accessible diagnostics, therapeutics, vaccines, and social measures.

Since late 2020, a number of VOCs have emerged that seriously affect global public health, including Alpha (PANGO lineage, B.1.1.7) in the United Kingdom, Beta (B.1.351) in South Africa, Gamma (P.1) in Brazil, and Delta (B.1.617.2) in India [2,5,6,7]. While Alpha, Beta, and Gamma VOCs disseminated globally at this time, it was the extremely transmissible Delta variant that ultimately displaced all of the other VOCs in most of the countries [8]. It is important to note that the Centers for Disease Control and Prevention (CDC) has recently shifted Alpha, Beta, Gamma, and Delta VOCs in the Variants Being Monitored (VBM) category. However, WHO has retained them in the VOCs category. In November 2021, while Delta was still causing significant levels of transmission in several countries, a new variant, B.1.1.529, was detected in a specimen collected on 9 November 2021 from South Africa and reported to WHO on 24 November 2021 [9]. The WHO named this variant as the Omicron variant—the fifth VOC of SARS-CoV-2 [10].

Omicron is distantly related to other VOCs, and it has dominated SARS-CoV-2 infections worldwide [11,12,13,14,15,16]; however, it has been found to produce less severe disease symptoms than previously documented VOCs [17,18]. Nonetheless, epidemiological evidence suggests that the Omicron VOC has a two- to three-fold greater risk of re-infection, which could be due to the capacity to circumvent the pre-existing immunity acquired by infections or vaccinations [19]. In comparison with the original Wuhan-Hu-1 reference strain, the Omicron VOC accumulated 53 mutations, with 30 mutations in the spike protein alone. This unusual preponderance of mutations, particularly in Omicron’s spike protein, has been demonstrated to enhance the transmissibility [20,21] as well as to escape from several neutralizing antibodies (NAbs) [22,23,24,25].

The Omicron VOC is undergoing substantial genetic evolution, as evidenced by the concurrent identification of three sub-lineages of Omicron (BA.1, BA.2, and BA.3) that are quite distinct from one another [9]. Initially, BA.1 proved to be the most prolific sub-lineage, spreading rapidly worldwide; however, BA.2 later surpassed BA.1 globally and emerged as the dominant sub-lineage. Recently, two more sub-lineages, BA.4 and BA.5, were discovered in South Africa [26]. Here, we focus especially on the BA.1 and BA.2 sub-lineages, which quickly spread concurrently in several countries throughout the world, and were the first recognized as Omicron VOCs.

Given the scarcity of studies decoding the origin and evolution of Omicron VOCs, we first deciphered and compared the unique mutational profiles of Omicron VOCs (BA.1 and BA.2 sub-lineages) to those of previously known VOCs (Alpha, Beta, Gamma, and Delta). Their functional characterization is also discussed in detail, which will improve our understanding of their clinical implications. Second, we aimed to explore the evolutionary dynamics of the SARS-CoV-2 Omicron VOC by utilizing root-to-tip regression analysis, recombination analysis, and Bayesian evolutionary analysis based on 32,170 whole genomes sampled globally between November 2021 and January 2022. Finally, we characterize the selective pressures that may have influenced the initial rapid evolution of the BA.1 and BA.2 sub-lineages.

## 2. Materials and Methods

### 2.1. Collation of SARS-CoV-2 Omicron Variant Complete Genome Dataset

The high-quality complete genome sequences of SARS-CoV-2 Omicron variant (*n* = 32,170) were obtained from the GISAID, which was deposited between November 2021 and January 2022 (http://gisaid.org/, accessed on 3 May 2021) [27]. These sequences were filtered out based on the following criteria: (1) low-quality sequences carrying unusual characters other than A, T, G, and C; (2) duplicate sequences with 100% nucleotide identity; and (3) sequences with incomplete associated information, such as sampling dates. Following filtration, the remaining high-quality sequences (BA.1 = 767 and BA.2 = 1002) were aligned using MAFFT v.7.490 [28], and subsequently, open reading frames (ORFs) were extracted manually from the aligned complete genomic sequences of the SARS-CoV-2 Omicron variant using BioEdit [29]. Therefore, the findings of this study are based on metadata associated with 1769 high-quality SARS-CoV-2 Omicron sequences available on GISAID from November 2021 to January 2022, via EPI_SET_221127gp (accessible at https://doi.org/10.55876/gis8.221127gp, accessed on 2 June 2021) (Table S1).

### 2.2. Recombination Analysis

The complete coding genomic sequences of SARS-CoV-2, where the ORFs were concatenated in the following order: ORF1ab + S + ORF3a + E + M + ORF6 + ORF7a + ORF7b + ORF8 + N + ORF10, were screened for recombination signals using RDP v4.101, which implements nine distinct algorithms: RDP, GENECONV, Bootscan, MaxChi, Chimaera, Siscan, PhylPro, LARD, and 3seq [30]. Using the default settings, these sequences were examined for each identified recombination breakpoint. In order to reduce false positive recombination signals, we only considered recombination events detected by at least two of the nine algorithms.

### 2.3. Root-to-Tip Regression Analysis to Assess the Temporal Signals

In order to assess the temporal signals in the dataset, we employed root-to-tip regression analysis on the entire coding genomic sequences of SARS-CoV-2 Omicron variant. Briefly, root-to-tip regression analyses are commonly used to estimate the relationship between root-to-tip genetic divergence and sampling dates generated from Maximum Likelihood (ML) phylogeny. The slope of the regression line provides an estimate of the evolutionary rates (substitutions per site per year), whereas the intercept with the time axis estimates the age of the root.

We first screened for the Maximum Likelihood (ML) fits of 88 alternative nucleotide substitution models for the SARS-CoV-2 Omicron sub-lineages’ (BA.1 and BA.2) datasets, and subsequently used ModelFinder to identify the best fitting nucleotide substitution model based on the Bayesian Information Criterion (BIC) (Table S2) [31]. The phylogenetic trees were then estimated using the ML inference and ultrafast bootstrap with 1000 replicates as implemented in IQ tree v2.1.2 [32]. Finally, these ML trees were used to investigate the temporal molecular evolutionary signals for each SARS-CoV-2 Omicron sub-lineage using TempEst v1.5.3 [33].

Of the complete dataset containing high-quality, unique Omicron BA.1 (*n* = 767) and BA.2 (*n* = 1002) complete genomic sequences, we removed outliers (often caused by sequencing errors or incorrect labeling) that did not fit to a root-to-tip regression and sequences showing evidence of recombination signals aside from that of the BA.1 (*n* = 381) and BA.2 (*n* = 579) dataset. As a result, after removing the outliers and recombinant sequences, we were left with 386 BA.1 and 423 BA.2 genomic sequences. Subsequently, to assess and improve the temporal signals, we generated three datasets by randomly picking up unique, representative Omicron sequences from each country: (i) one sequence from each sampling date (*n* = 26 for BA.1 and *n* = 48 for BA.2), (ii) two sequences from each sampling date (*n* = 75 for BA.1 and *n* = 86 for BA.2), and (iii) three sequences from each sampling date (*n* = 107 for BA.1 and *n* = 116 for BA.2).

### 2.4. Molecular Clock Phylogenetics

To infer the substitution/evolutionary rates and timescale of SARS-CoV-2 Omicron variant, Bayesian inference analyses were performed on the second dataset containing 161 dated, non-recombinant nucleotide sequences for the complete coding sequences of the BA.1 (*n* = 75) and BA.2 (*n* = 86) sub-lineages using a Markov Chain Monte Carlo (MCMC) framework [34], implemented in the Bayesian evolutionary analysis by sampling trees (BEAST) v2.6.7 [35]. As previously indicated, the best-fit nucleotide substitution model for each Omicron sub-lineage dataset was chosen.

To identify the best combination of tree priors and clock models, we tested and compared four coalescent tree priors: a constant population size [36], exponential population [37], Bayesian skyline [38], and extended Bayesian skyline [39] tree prior; and two clock models: a strict clock and an uncorrelated relaxed clock with log-normal distribution (UCLN) [40]. All Bayesian analyses were run for 100 million steps across two independent MCMC simulations with states and parameters sampled after every 10,000 steps. To find the best tree prior-clock model combination, Bayesian model testing, a statistical fit measure calculated by computing the log marginal likelihood, was performed and subsequently, each model combination was ranked accordingly. The log Bayes factor (BF) is the difference between two tree prior-clock models’ log marginal likelihoods [41]. A log BF of at least 1.1 in favor of a model is described as ‘substantial evidence’, with 2.3 being ‘strong’ and 4.6 being ‘decisive’ [42]. We considered two marginal likelihood estimators: path sampling and stepping-stone sampling [43,44,45]. The parameters for the best-fit model combination for Omicron sub-lineages attained an effective sample size of more than 200, indicating adequate sampling. Using Tracer v1.7.1, we extracted time to the most recent common ancestor (tMRCA) and clock rate estimations from the best-fit model combination [46]. After deleting the first 10% of samples as burn-in, the maximum clade credibility (MCC) tree was extracted using TreeAnnotator v1.8.4 [47]. The MCC trees were visualized using FigTree v1.4.4 (http://tree.bio.ed.ac.uk/software/figtree/, accessed on 11 July 2022). All these analyses were performed on the Hokusai BigWaterfall supercomputer of the Institute of Physical and Chemical Research (RIKEN), Japan.

### 2.5. Selection Pressure Analysis

The complete coding sequences of SARS-CoV-2 Omicron VOC were screened for the presence of recombination breakpoints using the Genetic Algorithm for Recombination Detection (GARD) method, implemented in the HyPhy package’s Datamonkey web server [48]. This screening was necessary because the inference based on the data with recombination breakpoints frequently yields more false positive sites [49,50]. In the cases of data with recombination breakpoints, the sequences were partitioned into the recombination blocks, and the selection pressure was estimated individually for each block. Then, using the Single Likelihood Ancestor Counting (SLAC) method, the site-specific selection pressure within the SARS-CoV-2 Omicron variant was estimated as the ratio of nonsynonymous (dN) to synonymous (dS) nucleotide substitutions per site (ω = dN/dS) [48].

We tested and compared the results of sites under a selection pressure estimated by five different methods with their default parameters, namely, the SLAC [48], Fixed Effect Likelihood (FEL), Fast Unbiased Bayesian AppRoximation (FUBAR) [51], adaptive Branch-Site Random Effects Likelihood (aBSREL) [52,53], and Branch-Site Unrestricted Statistical Test for Episodic Diversification (BUSTED) [54], all available at the Datamonkey web server. Furthermore, the sites in each ORF of SARS-CoV-2 Omicron variant experiencing positive/diversifying and negative/purifying selection pressure were taken into account only when anticipated by at least two of the aforementioned methods. 

## 3. Results and Discussion

### 3.1. Mutational Scanning of SARS-CoV-2 Omicron Reveals Its Independent Emergence

**Figure 1:**
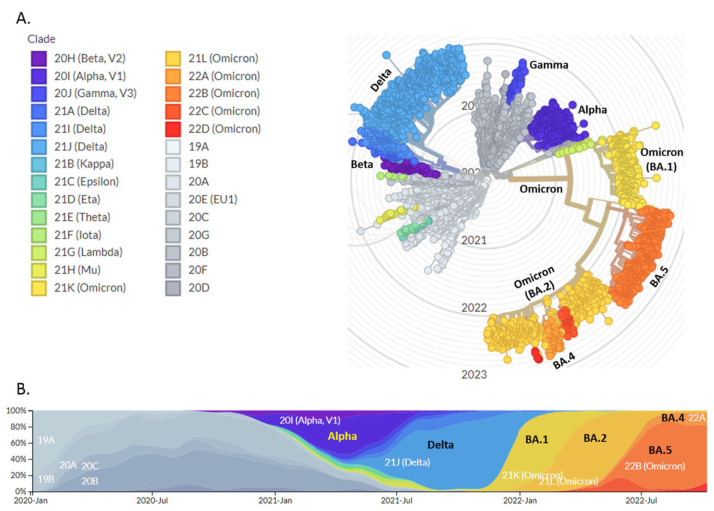
(**A**) A time-resolved maximum likelihood phylogeny of a representative global subsample of 3004 SARS-CoV-2 complete genomes sampled between December 2019 and October 2022 from the GISAID. The variants of concern (VOCs) are denoted by different colour schemes. The figure was generated by the Nextstrain using the data from the GISAID, (**B**) The global distribution frequencies of VOCs are denoted by different colour schemes over the timespan since SARS-CoV-2’s emergence.

**Figure 2:**
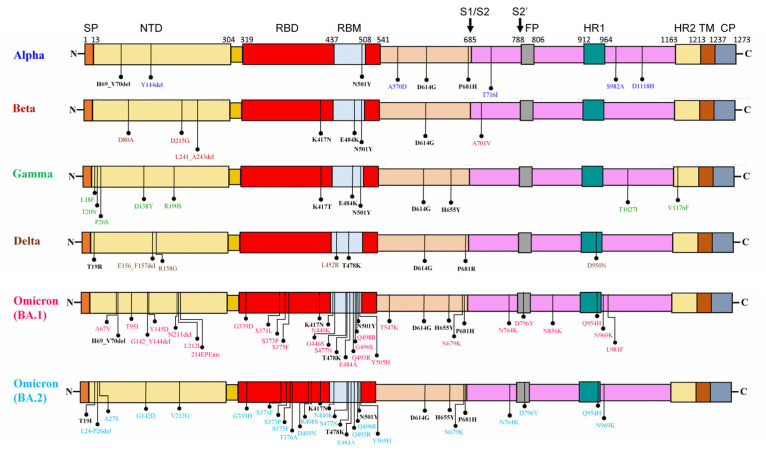
Mutational profiles of the Variants of Concern’s spike protein. Mutations that differed from those of other VOCs are denoted by different VOC-specific colours, while the shared mutations are represented by the black colour. SP, Signal Peptide; NTD, N-terminal domain; RBD, Receptor-Binding Domain; RBM, Receptor-Binding Motif; SD1, subdomain 1; SD2, subdomain 2; FP, Fusion peptide; HR1, Heptad repeat region 1; HR2, Heptad repeat region 2; TM, Transmembrane domain; CP, Cytoplasmic domain.

The recently discovered Omicron VOC is distantly related to earlier documented VOCs (Alpha, Beta, Gamma, and Delta) and has a remarkably high number of mutations in the spike protein (Figure 1A). As a result of Omicron’s continued genetic evolution, two sub-lineages (BA.1 and BA.2) were initially identified; BA.1 emerged as the dominant sub-lineage in late 2021 and spread quickly throughout the world, but BA.2 quickly overtook BA.1 on a global scale (Figure 1B). The identification of a highly divergent Omicron VOC advocated the possibility that Omicron may have evolved in a cellular micro-environment completely different from other known VOCs. In comparison to the wild-type SARS-CoV-2 (WT), Omicron’s receptor-binding domain (RBD) contains 15 mutations, 10 of which are in the receptor-binding motif (RBM), which mediates binding to host cells via the angiotensin-converting enzyme 2 (ACE2) receptor and to the majority of NAbs (Figure 2). Four mutations in Omicron’s RBM (N440K, S477N, T478K and N501Y), and one in RBD (G339D) have been demonstrated to improve binding affinity to human ACE2 [55,56].

Furthermore, 8 RBD mutations (G339D, S373P, S375F, N440K, E484A, Q493R, Q498R, and Y505H) have been shown to be associated with escape from a wide range of different classes of NAbs [57,58,59,60,61,62,63]. These RBD mutations, together with N-terminal domain (NTD) mutations, particularly H69–V70del and G142–Y144del, favor Omicron’s evasion from the majority of NAbs induced either by infections or vaccinations [22,64]. Notably, the mutation profile of Omicron BA.2′s NTD (T19I, L24-P26del, A27S, G142D, and V213D) differs significantly from that of BA.1, although their functional roles remain unknown (Figure 2).

Some mutations in the Omicron’s spike protein may also contribute to modulating the virus host spectrum. For example, the acquisition of positively charged amino acids at 493 and 498 (Q493K and Q498H) has been shown to allow SARS-CoV-2 to infect mice via interacting with murine ACE2 [65,66]. These two mutations, Q493R and Q498R (both containing positively charged amino acids and found in Omicron’s RBM), were acquired after 30 passages in the mouse lung (GISAID accession number EPI_ISL_1666328) [67]. Furthermore, H655Y was selected during replication in the mink model, implying a role in modifying the host range [68]. As a result, Q493R, Q498R, and H655Y carried by Omicron’s spike protein reflects its adaptation in mice and mink. These findings, together with a recent study demonstrating Omicron’s ability to mediate the enhanced entry into cells expressing multiple animal species’ ACE2 [69], imply that, Omicron may have a broader host range and a greater proclivity to establish an animal reservoir for its family than previously known VOCs.

Intriguingly, of 30 mutations in the Omicron’s spike protein, it shares only 8 mutations with other known VOCs, including H69-V70del and P681H in Alpha, K417N in Beta, H655Y in Gamma, T19I and T478K in Delta, N501Y in Alpha, Beta, and Gamma, and D614G in Alpha, Beta, Gamma, and Delta. Omicron’s unique mutation profile, combined with the amino acid substitution pattern’s low similarity to other known VOCs, opens the door for designating any of the previously reported VOCs or other variants as its most recent common ancestor. Furthermore, we noted distinct sub-lineage-specific mutations in the spike protein of BA.1 (A67V, T95I, G142-Y144del, Y145D, N211del, L212I, 214EPEins, S371L, G446S, T547K, N856K, and L981F) and BA.2 (T19I, L24-P26del, A27S, G142D, V213G, S371F, T376A, D405N, and R408S), possibly indicating their separate/independent emergence and evolution. Furthermore, regardless of the vaccination status, these sub-lineage-specific mutations may be associated with a higher susceptibility of infection by the BA.2 sub-lineage in comparison to the BA.1 sub-lineage [70].

Out of a cluster of three substitutions (H655Y, N679K, and P681H) found near the S1/S2 furin cleavage site of Omicron’s spike protein, two substitutions (P681H in Alpha, H655Y in Gamma, and P681R in Delta) have been demonstrated to facilitate cleavage of the spike protein and increase viral fusogenicity in the host cells [68,71,72,73]. Additionally, the spike protein’s fusion peptides of both Omicron’s sub-lineages (BA.1 and BA.2) contained a D796Y substitution that was absent from the previously identified VOCs. The combination of these substitutions may enhance the fusogenicity and transmissibility of Omicron [68].

Other than the spike glycoprotein, Omicron has several mutations in other proteins. The BA.1 sub-lineage is distinct from the BA.2 sub-lineage in that the former had three substitutions (K38R, L1266I, and A1892T) and one deletion (S1265 del) in nsp3, but the latter did not. The rest of the mutations are common in both the sub-lineages, including T492I in nsp4, P132H in nsp5, a S106-G107-F108 deletion and I189V in nsp6, P323L in NS12, and I42V in nsp14. Nonetheless, little is known about their functional roles, aside from: a deletion in nsp6 (del105–107) for the evasion of innate immunity [74], P323L in nsp12 (RNA-dependent RNA polymerase) for reduced binding affinity to remdesivir [75], and two mutations (R203K and G204R) in nucleocapsid for enhanced infectivity [76].

### 3.2. Recombination Analysis

Recombination is a fundamental mechanism for generating diversity among positive-sense RNA viruses, including SARS-CoV-2, and is an important tool for understanding the evolutionary history of viruses. Furthermore, the Bayesian molecular dating analyses on the dataset having evidence of recombination can result in the biased phylogenetic and phylodynamic inferences [77,78]. As a result, the construction of the dataset free from the recombination signals is a crucial step in deriving the molecular clock phylogenetics inferences.

We individually screened the sequences of the Omicron BA.1 and BA.2 sub-lineages for recombination signals using the RDP v4.101, which implements nine distinct algorithms to locate evidence of recombination signals. In the SARS-CoV-2 Omicron BA.1 sub-lineage dataset, we found a total of four recombination signals that were recognized by at least two different algorithms (Table S3). Three of these, however, lacked a high level of evidence. We found moderate evidence for only one recombination signal, which was identified by Chimaera (*p* = 0.0025), 3Seq (*p* = 0.0005), and Maxchi (*p* < 0.0001) in the NTD encoding region of spike protein. The breakpoint positions for this moderate recombinant signal were 21613 for the 5′ breakpoint and 27265 for the 3′ breakpoint. However, the recombination analyses could not identify the recombination signals in the Omicron BA.2 lineage dataset. The identified recombinant sequence, hCoV-19/env/Austria/CeMM21831/2022|EPI_ISL_9011265, detected in a wastewater sample from Austria on 2 January 2022, could have 78 minor and 14 major parental sequences. This recombinant sequence may have been derived from the major and minor parental sequences of the BA.1 sub-lineage (for example, BA.1.1, and BA.1.17). Since January 2022, multiple SARS-CoV-2 recombinants have been identified, including XBB (a recombinant of the BA.2.10.1 and BA.2.75 sub-lineages with a breakpoint in S1), XD (a recombinant of Delta and Omicron BA.1; S protein from BA.1 and the remainder from the Delta genome), XE (a recombinant of Omicron BA.1 and BA.2; spike and structural proteins from BA.2, and the remainder from BA.1), and XF (a recombinant of Delta and Omicron BA.1, spike and structural proteins from BA.2, and the remainder from the Delta genome), of which XBB finds its place in the Variants Under Monitoring (VUM), as classified by the WHO [4]. Therefore, the continuous monitoring of recombinants, especially in wastewater samples, together with combined individual testing is an effective and efficient approach in forecasting of new SARS-CoV-2 variants, thereby assisting the scientific community in preparing for future public health challenges. Lastly, we identified and removed all the recombinant sequences projected to convey even a low level of evidence from the dataset before conducting the Bayesian molecular dating analyses. 

### 3.3. Bayesian Molecular Dating Analyses of Omicron VOC

After screening 88 distinct nucleotide substitution models for the SARS-CoV-2 Omicron sub-lineages (BA.1 and BA.2) datasets, General Time Reversible (GTR + F + I) and Tamura-Nei (TN + F + I) models were found to be the best nucleotide substitution models for BA.1 and BA.2 sub-lineages, respectively (Table S2). The ML trees were generated using these best fitting nucleotide substitution models. Using TempEst v1.5.3, root-to-tip regression analysis was performed on the ML trees generated separately for the Omicron BA.1 and BA.2 sub-lineages to assess the temporal molecular evolutionary signals. The coefficient of the determinant, R^2^, which measures the clock-likeness of the sequences, and the correlation of coefficient (r) were low for the BA.1 (r = 0.371 and R^2^ = 0.137) and BA.2 (r = 0.166 and R^2^ = 0.027) datasets.

**Figure 3:**
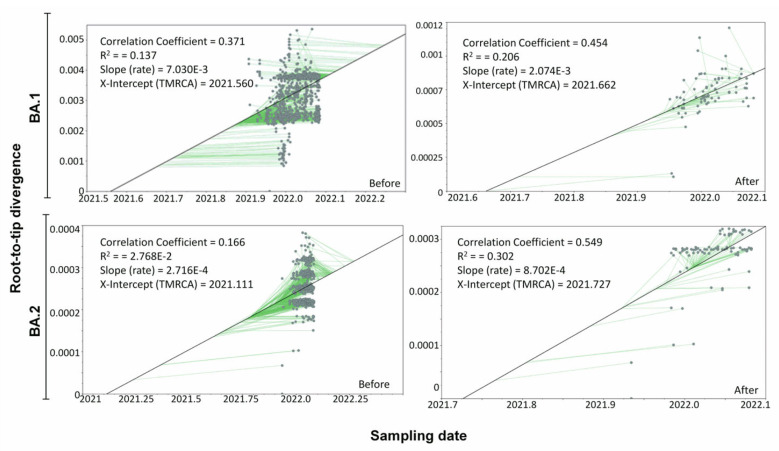
Root-to-tip regression analyses on the maximum likelihood (ML) trees generated for original dataset comprising Omicron BA.1 and BA.2 sub-lineages (Before) and dataset after removing the outliers (After).

To improve the temporal signals in our dataset, we first identified and removed any outliers (often caused by sequencing errors or incorrect labeling) that did not fit to a root-to-tip regression. Subsequently, while maintaining the sequence heterogeneity, we generated three datasets by randomly picking up unique Omicron sequences from each country: (i) one sequence from each sampling date, (ii) two sequences from each sampling date, and (iii) three sequences from each sampling date. By doing this, temporal signals for both Omicron sub-lineages BA.1 (r = 0.454 and R^2^ = 0.206) and BA.2 (r = 0.549 and R^2^ = 0.302) improved significantly in the second set of data (Figure 3, Table S4).

**Figure 4:**
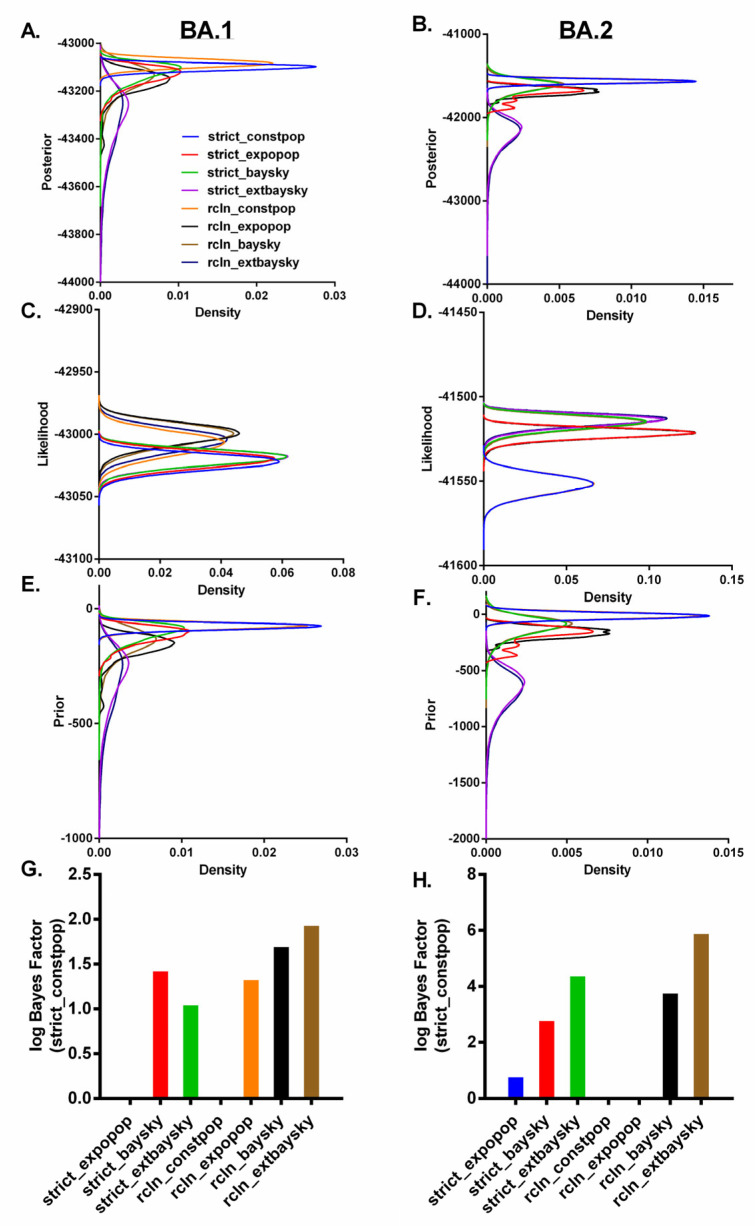
Bayesian hypothesis testing using the log Bayes factor. Posterior (**A**,**B**), Likelihood (**C**,**D**) and Prior (**E**,**F**) estimates for comparative evaluation of eight combinations of tree priors and clock models implemented in BEAST v2.6.7 are shown for the BA.1 and BA.2 sub-lineages, respectively. (**G**,**H**) Bayesian hypothesis testing using the log Bayes factor. The best fit combination of tree priors and clock models was the constant population coalescent tree prior with strict clock for both the Omicron sub-lineages, BA.1 and BA.2.

Next, Bayesian molecular dating analyses were performed on the second dataset containing 161 dated, non-recombinant nucleotide sequences for the complete coding sequences of Omicron. We compared the prior, posterior, and likelihood distributions of each of the eight tree priors-clocks combinations in order to determine the best model-fit for the Omicron sub-lineages (Figure 4A–F). The estimated tMRCA dates and evolutionary rates of BA.1 sub-lineage were relatively comparable across all the tree priors (e.g., constant population size, exponential population, Bayesian skyline, and extended Bayesian skyline), but varied greatly depending on the clock model used. Comparatively, the estimated tMRCA dates and evolutionary rates of BA.2 sub-lineage were very similar depending on the clock model used, but varied across different tree priors (Table S5).

**Figure 5:**
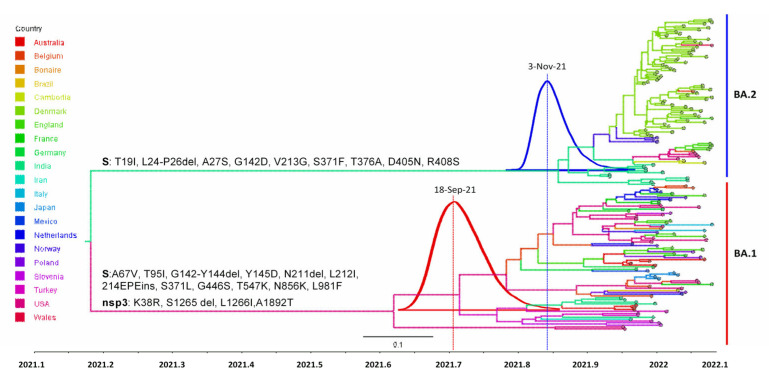
Time-resolved maximum clade credibility phylogeny of the Omicron BA.1 and BA.2 sub-lineages (*n* = 161, sampled between November 2021 and January 2022). Mutations that characterize Omicron sub-lineages are indicated on the respective branches. The posterior distribution of the tMRCA for each sub-lineage is also shown. The countries as a trait for each sample are denoted by distinct colours.

The Constant Population coalescent tree prior with strict clock was the best fit to both of the Omicron sub-lineages, according to Bayesian hypothesis testing using the log Bayes factor (Figure 4G-H). A time-scaled maximum-clade-credibility tree showed that all of the omicron sequences could be separated into two distinct sub-lineages, BA.1 (*n* = 75) and BA.2 (*n* = 86). The most recent common ancestors (tMRCA) for the BA.1 and BA.2 sub-lineages sequences were estimated to be 18 September 2021 (95% highest posterior density (HPD) 4 August–22 October 2021) and 03 November 2021 (95% HPD 26 September–28 November 2021), respectively (Figure 5). The substitution rates of BA.1 and BA.2 were estimated to be 1.435 × 10^−3^ (95% HPD  =  1.021 × 10^−3^ − 1.869 × 10^−3^) substitution/site/year and 1.074 × 10^−3^ (95% HPD  =  6.444 × 10^−4^ − 1.586 × 10^−3^) substitution/site/year, respectively, which is in line with several previous studies that estimated the substitution rates of SARS-CoV-2 [9,79,80,81].

In comparison to our dataset’s tMRCA for the BA.1 sub-lineage, the estimated tMRCA of the South African BA.1 sub-lineage sequence was found to be early October 2021 (9 October 2021, 95% HPD 30 September–20 October 2021) [9]. This discrepancy is likely due to differences in the dataset’s geographical heterogeneity. The tMRCA estimate for the BA.2 sub-lineage is in perfect accord with other studies, such as the tMRCA of BA.2 sub-lineage on 6 November 2021 (95% HPD = 9 October 2021 to 29 November 2021) [26], and mid-November for the Philippines BA.2 lineage sequences (18 November 2021; 95 % HPD = 6–28 November 2021) [82]. Since the BA.1 and BA.2 sub-lineages exhibit different tMRCAs, which is in line with the more recent emergence of BA.2 as compared to BA.1, they are expected to bear sub-lineage specific mutational profiles, and it is possible that these two sub-lineages of Omicron VOC might have originated and evolved independently. 

The evolutionary history of Omicron is presently governed by three hypotheses. The first hypothesis is that Omicron might have spread silently in a geographical region with limited surveillance and sequencing facility [83,84]. Second, Omicron might have evolved in an immunocompromised patient, allowing long-term sustained evolution and adaptation of the virus [83,85]. Third, the Omicron might have accumulated mutations in a non-human host before jumping to humans [85,86]. Presently, the second hypothesis seems to be the more plausible explanation for the evolutionary origin of Omicron [86]. Nonetheless, in the event of the emergence of multiple new mutations in the Omicron’s spike protein, which are quite distinct in the BA.1 and BA.2 sub-lineages, as well as their estimated separate most recent common ancestor, it may be more plausible to conclude that a combination of RBD- and NTD-directed classes of antibody therapeutics at sub-optimal doses in COVID-19 patients or optimal doses in an immunocompromised patient or waned vaccine-induced immunity may have provided a conducive environment to accumulate multiple mutations in Omicron’s spike protein. However, the role of intermediate hosts, particularly rodents, in Omicron transmission and evolution, followed by reverse-zoonosis, should not be neglected. 

### 3.4. Selection Analysis

The rapid evolution of RNA viruses, particularly SARS-CoV-2, is governed by increasing and persistent selection pressure, leading to the creation of viruses with altered genetic and phenotypic characteristics [87,88]. These evolutionary changes are the result of two opposing forces: positive selection (which generates various genetic changes beneficial for virus enhanced fitness to its host) and negative selection (which aims to maintain fitness without producing any advantageous changes). Therefore, positive selection may have accelerated the accumulation of multiple mutations in the Omicron BA.1 and BA.2 sub-lineages. To test for evidence of selection (both positive/diversifying and negative/purifying) in the Omicron’s evolution, we employed a selection pressure estimation pipeline comprising of five methods: SLAC, FEL, FUBAR, aBSREL, and BUSTED at the Datamonkey web server of the HyPhy package. We filtered out sequences that do not have Omicron’s spike protein signature mutational profile and then extracted individual protein coding regions (11 ORFs) from unique BA.1 (*n* = 689) and BA.2 (*n* = 948) sub-lineages to estimate the site-specific selection pressure. To improve the robustness of site-selection and reduce the false positive rates, only sites predicted by at least two of the aforementioned methods were considered. 

Among the 11 ORFs, three ORFs (ORF3a, ORF7a, and ORF7b) experienced a strong selection (ratio of non-synonymous to synonymous substitutions is more than 1.5). There were seven positively selected sites in ORF1ab, 13 in the spike protein, and 1 in the Nucleocapsid protein of the Omicron BA.1 sub-lineage, and only one in the Membrane protein of BA.2 sub-lineage, indicating that multiple codon sites drive the genetic diversity in the BA.1 sub-lineage (Table 1). Furthermore, of the spike protein’s 13 sites in the BA.1 sub-lineage, eight sites (339, 371, 375, 440, 446, 484, 493, and 505) that showed evidence of diversifying or positive selection, were associated with escape from different classes of NAbs [57,58,59,60,61,62,63], implying that these sites are still evolving in order to modify BA.1′s neutralization profile. Importantly, four sites in S (346, 452, 554, and 1260), and one each in N (215) and nsp6 (3646) that showed positive selection signals were not Omicron-defining mutations, and these sites could have been carried by its most recent common ancestor. However, three sites carrying the positive selection signals converge on mutations found in previously identified SARS-CoV-2 VOCs (S:452, N:215, and nsp6:3646 in Delta; S:346 in Mu VOI). These observations support the notion that Omicron might have originated and evolved independently.

## 4. Conclusions

The findings of this study, which investigated the early global evolutionary dynamics of the recently identified, highly divergent SARS-CoV-2 Omicron VOC sampled between November 2021 and January 2022, combined with mutational profiling suggest that the Omicron BA.1 and BA.2 sub-lineages originated independently and evolved over time. The currently available evidence supports the idea that the Omicron VOC may have originated due to the long-term persistence in an immunocompromised patient or COVID-19 patient with waned vaccine-induced immunity. However, the role of intermediate hosts, particularly rodents, in Omicron transmission and evolution, followed by reverse-zoonosis, should not be neglected. This study also advocates the continued diversifying selection that may alter the neutralization profile of BA.1. Numerous mutations, particularly in the spike protein’s NTD and nsp3, whose functions are still unclear, warrant functional characterization in order to understand their contributions to differential viral transmissibility and diminished efficacy of therapeutics and vaccines. Finally, this study emphasizes the significance of ongoing global genomic surveillance, Bayesian molecular dating analyses, and mutational profiling in understanding the virus’s evolutionary dynamics and, as a result, mitigating the impact of emerging variants on public health.

## Figures and Tables

**Table 1:** Amino acid sites under diversifying or purifying selection for each protein-encoded region of the Omicron BA.1 and BA.2 sub-lineages.

	BA.1			BA.2		
ORFs	dN/dS	Diversifying Selection	Purifying Selection	dN/dS	Diversifying Selection	Purifying Selection
ORF1ab	0.580	nsp3 (2710); nsp4 (3255); nsp5 (3395); nsp6 (3646, 3758); nsp12 (5063); nsp14 (5967)	nsp2 (341, 735); nsp3 (1120, 1707, 1750, 1903, 2676); nsp4 (2907); nsp5 (3290, 3458); nsp6 (3689); nsp10 (4310); nsp12 (4992); nsp13 (5444, 5541)	0.525	-	nsp2 (440); nsp3 (924, 1707, 2470, 2551); nsp4 (3100, 3245); nsp5 (3271, 3311); nsp13 (5616, 5746); nsp15 (6566); nsp16 (6819)
S	0.981	RBD (339, 346, 371, 375, 440, 446, 452, 484, 493, 505); SD1/SD2 (554); S2 (796, 1260)	SP (11); SD1/SD2 (543); S2 (1146)	0.653	-	NTD (296); RBD (336, 410)
ORF3a	2.040	-	-	1.266	-	-
E	0.401	-	-	0.341	-	67, 68
M	0.201	-	135	0.477	3	-
ORF6	0.259	-	-	0.597	-	-
ORF7a	1.52	-	-	1.91	-	-
ORF7b	1.67	-	-	1.37	-	-
ORF8	0.563	-	-	0.986	-	-
N	0.812	215	73, 324	0.880	-	329
ORF10	0.319	-	-	0.970	-	-

## Data Availability

Not applicable.

## Supplementary Materials

The following supporting information can be downloaded at: https://www.mdpi.com/article/10.3390/v14122764/s1, Table S1: An EPI_SET Identifier for the metadata of high-quality SARS-CoV-2 Omicron genomic sequences used in this study; Table S2: Identification of best fitting nucleotide substitution model based on the Bayesian Information Criterion (BIC) using the ModelFinder; Table S3: The recombination signals detected in the Omicron BA.1 sub-lineage; Table S4: Temporal signals in three datasets (of BA.1 and BA.2 sub-lineages) generated by randomly picking up unique Omicron sequences from each country: (i) one sequence from each sampling date (1SEQ), (ii) two sequences from each sampling date (2SEQ), and (iii) three sequences from each sampling date (3SEQ); Table S5: A comparison of the estimated tMRCA dates and evolutionary rates of Omicron’s BA.1 and BA.2 sub-lineages across all the tree priors and clock models combinations.
