# Supplementary material for: Bayesian Molecular Dating Analyses Combined with Mutational Profiling Suggest an Independent Origin and Evolution of SARS-CoV-2 Omicron BA.1 and BA.2 Sub-Lineages
Source: Viruses. 2022 Dec 12;14(12):2764. doi: 10.3390/v14122764 (PMC9788409; doi:10.3390/v14122764)
Supplement: Supplementary file 1 [file viruses-14-02764-s001.zip › Table S5.pdf]

Table S4a. Tree height statistics for the four tree priors and two clock models combinations of Omicron's BA.1 sub-lineage

Tree Height (Summary Statistic)	strict_constpop	strict_expopop	strict_baysky	strict_extbaysky	rcln_constpop	rcln_expopop	rcln_baysky	rcln_extbaysky
mean	0.3785	0.5146	0.797	0.7841	0.4201	1.4767	1.6195	1.2767
stderr of mean	1.07E-03	0.0297	0.1036	0.0672	3.30E-03	0.5896	0.1848	0.1961
stdev	0.0578	0.2936	3.6887	2.4421	0.0963	3.4091	5.0846	6.601
variance	3.35E-03	0.0862	13.6063	5.9638	9.28E-03	11.6221	25.8533	43.5737
median	0.3715	0.4217	0.461	0.4636	0.4021	0.6723	0.7344	0.4892
mode	n/a	n/a	n/a	n/a	n/a	n/a	n/a	n/a
geometric mean	0.3743	0.4655	0.5448	0.5439	0.4105	0.8025	0.866	0.6133
95% HPD Interval	[0.2758, 0.4943]	[0.2365, 1.1182]	[0.2313, 1.6447]	[0.2264, 1.6875]	[0.2681, 0.6107]	[0.2114, 4.13]	[0.1986, 4.5816]	[0.195, 3.0942]
auto-correlation time (ACT)	31025.27	9.24E+05	71002.23	68195.55	1.05E+05	2.69E+06	1.19E+05	79444.32
effective sample size (ESS)	2900.894	97.4153	1267.58	1319.749	853.6177	33.4318	756.6193	1132.882

Table S4b. Clock rate statistics for the four tree priors and two clock models combinations of Omicron's BA.1 sub-lineage

Clock rate (Summary Statistic)	strict_constpop	strict_expopop	strict_baysky	strict_extbaysky	rcln_constpop	rcln_expopop	rcln_baysky	rcln_extbaysky
mean	1.44E-03	8.50E-04	8.65E-04	8.72E-04	1.55E-03	4.20E-04	5.41E-04	8.03E-04
stderr of mean	4.97E-06	3.60E-05	2.96E-05	2.95E-05	1.11E-05	2.70E-05	4.40E-05	4.18E-05
stdev	2.17E-04	3.88E-04	4.17E-04	3.95E-04	2.92E-04	2.73E-04	4.15E-04	4.58E-04
variance	4.71E-08	1.51E-07	1.74E-07	1.56E-07	8.54E-08	7.45E-08	1.72E-07	2.09E-07
median	1.44E-03	8.39E-04	8.84E-04	8.92E-04	1.52E-03	3.72E-04	4.39E-04	7.80E-04
mode	n/a	n/a	n/a	n/a	n/a	n/a	n/a	n/a
geometric mean	1.43E-03	7.42E-04	7.24E-04	7.41E-04	1.52E-03	3.19E-04	3.82E-04	6.17E-04
95% HPD Interval	[1.0214E-3, 1.8697E-3]	[1.3571E-4, 1.5385E-3]	[9.1338E-5, 1.586E-3]	[9.3829E-5, 1.5625E-3]	[9.9915E-4, 2.13E-3]	[6.1752E-6, 9.2324E-4]	[1.6754E-6, 1.4072E-3]	[1.5313E-6, 1.5826E-3]
auto-correlation time (ACT)	47266.14	7.75E+05	4.53E+05	5.04E+05	1.30E+05	8.82E+05	1.01E+06	7.52E+05
effective sample size (ESS)	1904.133	116.1955	198.5462	178.4601	693.2781	102.0579	89.0793	119.6824

Table S4c. Tree height statistics for the four tree priors and two clock models combinations of Omicron's BA.2 sub-lineage

Tree Height (Summary Statistic)	strict_constpop	strict_expopop	strict_baysky	strict_extbaysky	rcln_constpop	rcln_expopop	rcln_baysky	rcln_extbaysky
mean	0.251	1.6815	3.0339	5.5056	0.2527	0.9229	4.0885	23.1817
stderr of mean	1.04E-03	0.4086	0.4105	2.0292	1.35E-03	0.1133	1.587	13.9612
stdev	0.0473	2.0952	17.4212	108.2619	0.0487	0.6587	53.574	619.6183
variance	2.24E-03	4.39	303.497	11720.64	2.37E-03	0.4338	2870.169	3.84E+05
median	0.2432	0.7633	0.9399	1.1497	0.2446	0.745	0.9077	1.2517
mode	n/a	n/a	n/a	n/a	n/a	n/a	n/a	n/a
geometric mean	0.2469	1.0069	1.165	1.4182	0.2485	0.776	1.1162	1.654
95% HPD Interval	[0.1735, 0.3462]	[0.2353, 6.6529]	[0.1744, 7.8034]	[0.1834, 9.9067]	[0.1724, 0.3492]	[0.2345, 2.0267]	[0.1541, 7.2364]	[0.1685, 16.6044]
auto-correlation time (ACT)	43163.32	3.42E+06	49980.18	31619.41	68969.36	2.66E+06	78976.81	45692.72
effective sample size (ESS)	2085.127	26.2906	1800.734	2846.385	1304.942	33.8241	1139.588	1969.701

Table S4d. Clock rate statistics for the four tree priors and two clock models combinations of Omicron's BA.2 sub-lineage

Clock rate (Summary Statistic)	strict_constpop	strict_expopop	strict_baysky	strict_extbaysky	rcln_constpop	rcln_expopop	rcln_baysky	rcln_extbaysky
mean	1.10E-03	8.85E-05	1.14E-04	9.67E-05	1.10E-03	9.58E-05	1.16E-04	9.34E-05
stderr of mean	6.41E-06	1.05E-05	3.10E-06	1.37E-06	8.62E-06	7.59E-06	2.66E-06	1.87E-06
stdev	2.45E-04	6.27E-05	8.61E-05	7.25E-05	2.50E-04	5.28E-05	8.42E-05	7.29E-05
variance	5.99E-08	3.93E-09	7.41E-09	5.25E-09	6.26E-08	2.79E-09	7.10E-09	5.32E-09
median	1.07E-03	8.09E-05	9.85E-05	8.26E-05	1.08E-03	8.49E-05	1.03E-04	7.88E-05
mode	n/a	n/a	n/a	n/a	n/a	n/a	n/a	n/a
geometric mean	1.07E-03	6.23E-05	7.92E-05	6.64E-05	1.07E-03	8.11E-05	8.26E-05	5.93E-05
95% HPD Interval	[6.4442E-4, 1.5862E-3]	[4.1855E-6, 2.0656E-4]	[8.6959E-8, 2.5982E-4]	[8.8487E-9, 2.2906E-4]	[6.3575E-4, 1.5991E-3]	[1.073E-5, 1.9392E-4]	[2.0722E-8, 2.6045E-4]	[1.4609E-9, 2.2732E-4]
auto-correlation time (ACT)	61770.57	2.53E+06	1.17E+05	32094.56	1.07E+05	1.86E+06	89842.29	59451.61
effective sample size (ESS)	1457.021	35.6289	771.458	2804.244	843.8335	48.3673	1001.767	1513.853
